# Periportal Right Colic Artery Arising From the Ventral Aspect of the Superior Mesenteric Artery With Associated Vascular Anomalies: A Case Report

**DOI:** 10.7759/cureus.111569

**Published:** 2026-06-26

**Authors:** Vinay Sharma, Padamjeet Panchal, CS Ramesh Babu

**Affiliations:** 1 Anatomy, Muzaffarnagar Medical College, Muzaffarnagar, IND; 2 Anatomy, All India Institute of Medical Sciences Patna, Patna, IND

**Keywords:** cadaveric dissection, complete mesocolic excision, ct angiography, d3 lymphadenectomy, middle colic artery, middle colic vein, right colic artery, superior mesenteric artery, superior mesenteric vein, vascular variation

## Abstract

The right colic artery (RCA) classically arises from the right convex surface of the superior mesenteric artery (SMA), crossing the superior mesenteric vein (SMV) anteriorly or posteriorly. We report a previously undescribed combination of three concurrent vascular anomalies identified during routine cadaveric dissection: an RCA arising from the ventral SMA surface with a periportal course producing visible indentation of the anterior SMV wall; an aberrant middle colic artery (MCA) arising from the ventral-left SMA surface with immediate bifurcation into right and left branches, the latter running along the inferior pancreatic border; and bifid middle colic venous drainage without a common trunk, with left tributaries draining to the splenic vein and right tributaries draining independently to the SMV.

The periportal RCA lies directly within the D3 (third level lymph node dissection) field, the central lymphadenectomy zone extending from the ileocolic artery (ICA) root to the MCA root along the SMA, where the surgeon skeletonizes the SMV and ligates colic vessels at their origins during laparoscopic right hemicolectomy with complete mesocolic excision (CME) and central vascular ligation (CVL). Unlike a standard anteriorly crossing RCA, which maintains a tissue plane between itself and the SMV, this periportal variant contacts the anterior SMV wall directly at the outset of central dissection, creating an unannounced risk of inadvertent SMV laceration during the medial-to-lateral approach. The independent venous drainage to the splenic vein cannot be controlled by standard middle colic vein (MCV) ligation. Preoperative high-resolution computed tomography colonography-angiography (CTC-A) is essential for anticipating such variants.

This case demonstrates that right colonic vascular anomalies may occur in complex combinations affecting both the arterial and venous systems. Detailed preoperative vascular mapping is strongly recommended before complete mesocolic excision/central lymphadenectomy to minimize the risk of catastrophic intraoperative hemorrhage.

## Introduction

The superior mesenteric artery (SMA) gives rise to three principal colic branches: the ileocolic artery (ICA), the right colic artery (RCA), and the middle colic artery (MCA). While the ICA and MCA are highly consistent vessels, present in 99.7% (95% CI 99.4%-99.8%) and 96.9% (95% CI 94.2%-98.8%) of individuals, respectively, in a meta-analysis of 4,691 patients across 41 studies, the RCA is the most variable, with a pooled prevalence estimate of 72.6% (95% CI 61.3%-82.5%) and extreme inter-study heterogeneity (I² = 98%) [1].

The RCA inconstantly reflects genuine anatomical variation: it may arise independently from the SMA, share a common trunk with the MCA (Yada type 2, ~18%) or ICA (Yada type 3, ~13%), or be entirely absent (~27%) [1-4]. When present, it most commonly runs anterior to the SMV (~87-89%), with posterior (retro-SMV) crossing in approximately 11% of cases [1,5,6]. Its origin is classically described from the right convex surface of the SMA; origin from the ventral (anterior) face has not been described as a distinct variant in any major classification system [2,7,8].

Complete mesocolic excision (CME) with central vascular ligation (CVL), first formalized by Hohenberger et al. in 2009, and the equivalent Japanese D3 (third-level lymph node dissection) lymphadenectomy require systematic skeletonization of the SMV and sequential ligation of the colic pedicles at their SMA origins [9]. Preoperative CT angiography and CT colonography-angiography (CTC-A) have become integral to planning these procedures, particularly given the high variability of right colonic vascular anatomy [10,11]. A ventral SMA origin with periportal SMV compression, as described here, represents a previously undescribed hazard within this dissection field.

The middle colic vein (MCV) is almost always present and typically drains into the SMV directly (~68-94%) or into the gastrocolic trunk of Henle (GCT), with rare drainage to the splenic vein, inferior mesenteric vein (IMV), or jejunal veins [12-14]. Absence of a common MCV trunk with divergent drainage to the splenic vein and SMV has not been reported concurrently with the arterial anomalies described here.

We present this case to document the variant, contextualize it within current literature, and highlight its surgical implications for laparoscopic right hemicolectomy with CME.

## Case presentation

Specimen details

A formalin-fixed male cadaver, approximately 35 years old, was studied during routine undergraduate anatomy instruction in our department. No history of previous abdominal surgery or vascular disease was identified on external or internal inspection. Dissection was performed by medical students under faculty supervision in accordance with institutional ethical guidelines and applicable human tissue regulations. Photographs and measurements were recorded at each stage.

Dissection findings

Following removal of the small bowel and mobilization of the greater omentum and transverse mesocolon, the root of the SMA was exposed from its aortic origin to the level of the ileocecal junction. The SMV was identified in its usual position to the right of and anterior to the SMA in its proximal segment. Three concurrent anomalies were documented:

Periportal Right Colic Artery

The RCA was identified arising from the ventral (anterior) surface of the SMA at a level between the origins of the MCA and ICA. Unlike the typical origin from the right convex surface. After its origin, the vessel coursed anterior to the SMV from left to right, creating a visible indentation of the anterior venous wall consistent with parietal compression and a periportal course. The RCA then continued laterally and somewhat inferiorly to supply the ascending colon and the right (hepatic) colic flexure. No posterior (retro-SMV) component was observed. This configuration is distinct from both the standard anterior crossing (where the RCA crosses in front of but away from the SMV) and the retro-SMV course (~11% of cases) documented in the literature [1,5].

Aberrant Middle Colic Artery

The MCA arose from the ventral and left aspects of the SMA, a configuration that differs from the anterior midline origin most commonly described. Immediately upon its origin, the MCA divided into right and left branches without a common trunk. The right branch ascended superiorly towards the right to supply the right transverse colon, with potential overlap with the RCA supply territory at the hepatic flexure. The left branch descended to run along the inferior border of the pancreas and distributed to the central and left transverse colon, a course anatomically consistent with the accessory middle colic artery (aMCA) described by multiple imaging-based studies [10,15].

Bifid Middle Colic Venous Drainage Without a Common Trunk

**Figure 1:**
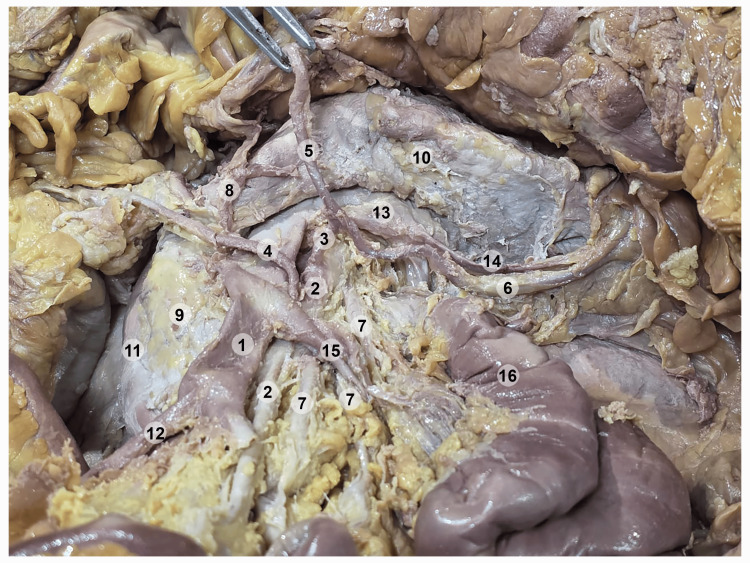
Cadaveric dissection of the right colon showing periportal right colic artery arising from the ventral aspect of the superior mesenteric artery, associated with aberrant middle colic artery origin and bifid middle colic venous drainage. Anterior view of the infra-mesocolic floor following removal of the small bowel and mobilization of the transverse mesocolon. 1: Superior mesenteric vein (SMV), 2: Superior mesenteric artery (SMA), 3: Middle colic artery (MCA) — with immediate bifurcation, 4: Right colic artery (RCA) — arising from ventral aspect of SMA, 5: Right branch of MCA, 6: Left branch of MCA (running along inferior pancreatic border), 7: Jejunal arteries, 8: Right side tributary of colic vein (RCV), 9: Head of pancreas 10: Body of pancreas, 11: Duodenum, 12: Ileocolic vein (ICV), 13: Splenic vein, 14: Left side tributary of MCA vein (draining into splenic vein), 15: Jejunal vein, 16: Jejunum.

The venous drainage accompanying the MCA branches was markedly abnormal. Normally, tributaries associated with the right and left MCA branches converge to form a single common MCV trunk that drains into the SMV [12,16]. In this specimen, the left tributaries (accompanying the left MCA branch running along the inferior pancreatic border) drained directly into the splenic vein. The right tributaries drained independently into the SMV. No common MCV trunk was formed. This represents a pattern of bifid divergent venous drainage into two separate divisions of the portal system (Figure 1).

**Figure 2:**
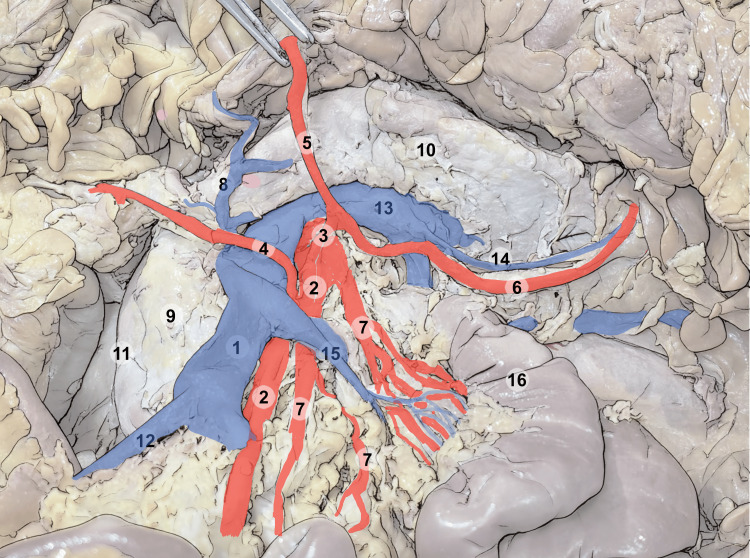
Colour-coded schematic overlay of the cadaveric dissection demonstrating the periportal right colic artery arising from the ventral aspect of the superior mesenteric artery with associated vascular anomalies. Arteries are highlighted in red and veins in blue. 1: Superior mesenteric vein (SMV), 2: Superior mesenteric artery (SMA), 3: Middle colic artery (MCA) — with immediate bifurcation, 4: Right colic artery (RCA) — arising from ventral aspect of SMA, 5: Right branch of MCA, 6: Left branch of MCA (running along inferior pancreatic border), 7: Jejunal arteries, 8: Right side tributary of colic vein (RCV), 9: Head of pancreas 10: Body of pancreas, 11 Duodenum, 12 Ileocolic vein (ICV), 13: Splenic vein, 14: Left side tributary of MCA vein (draining into the splenic vein), 15: Jejunal vein, 16: Jejunum. Red: Arterial structures, Blue: Venous structures Illustration created by Mr. Chandan Kumar (Artist in the Department of Anatomy). The illustration was created using Adobe Photoshop 2026 (Adobe Systems Software Ireland Ltd., Dublin, Ireland).

The color-coded schematic overlay illustrates the three concurrent vascular anomalies identified at dissection (Figure 2).

The ICA was present, arose from the right convex surface of the SMA, and followed a standard course posterior to the SMV before reaching the ileocecal region. The right and left tributaries of MCV were identified at the inferior border of the pancreas, entering the SMV and splenic vein, respectively, but were not further characterized. No additional colic or mesenteric arterial anomalies were identified.

## Discussion

Classification of RCA origin and why ventral origin is distinct

Existing classification systems for right colonic arterial anatomy, including those of Gamo et al. (2016), the Yada classification, and the 2021 Cirocchi meta-analysis, address the presence or absence of the RCA and the identity of the parent vessel (SMA, ICA, MCA, or common trunk) but do not classify the surface of the SMA from which the RCA arises [1,4,6-8]. In clinical practice, the SMA can be conceptually divided into four surfaces: right convex (typical site of ICA, RCA, and MCA origin), left concave (site of jejunoileal branches), anterior/ventral, and posterior/dorsal. A ventral origin places the RCA in direct proximity to the SMV immediately at its origin, fundamentally altering its spatial trajectory.

In Gamo et al.'s cadaveric and CT study of 50 and 560 subjects, respectively, Pattern I (independent origin of all three colic branches) was found in 40% cadaveric and 73.69% CT samples, with the RCA as the principal source of variation across all patterns [3,7]. Cirocchi et al. (2021) confirmed that studies using a strict SMA-only origin definition for the RCA reported a higher prevalence (82.9%) compared to those using a function-based definition (53.7%), highlighting the definitional sensitivity [1]. None of these or any other study to our knowledge specifically categorizes origin from the ventral SMA face.

The Yada classification's Type 1 (independent SMA origin) would technically include a ventral origin, as would Gamo's Pattern I. However, these classifications group all independent origins regardless of the SMA face from which the vessel arises, thereby obscuring the clinically critical periportal relationship created by a ventral origin. We suggest that future classifications include the SMA surface of origin (right convex, left concave, ventral, or dorsal) as an additional descriptor, particularly as high-resolution CTC-A is now capable of visualizing this level of anatomical detail [3,10,11,17].

The periportal course and SMV indentation

The most surgically significant feature of the present case is the periportal compression of the anterior SMV wall by the RCA. In standard anterior SMV crossing, the RCA passes in front of the vein with a normal tissue plane between them. A ventral SMA origin leaves effectively no space between the RCA's origin and the anterior SMV wall, so the vessel must either compress the vein (as observed here) or take an abrupt lateral deflection. The indentation visible on gross inspection indicates that the vessel was under some tension against the venous wall.

Murono et al. (2016) demonstrated in 536 patients undergoing 3D-CT angiography that the RCA ran ventral to the SMV in 89.4% and posterior to the SMV in 10.6% of cases; they further showed that when the RCA ran posterior to the SMV, the ICA also ran posterior in all cases, suggesting linked embryological determinants [5]. Cirocchi et al. (2021) confirmed the majority anterior course (87.3%) and noted that inadvertent traction causing SMV injury is a recognized and potentially fatal complication of right hemicolectomy [1,14]. A periportal RCA exerting direct pressure on the anterior SMV wall represents an extreme and qualitatively different risk from either standard anterior crossing or the posterior (retro-SMV) course. In the standard anterior crossing, the RCA maintains a definable tissue plane between itself and the SMV, allowing the surgeon to develop this plane safely during medial-to-lateral dissection. In the retro-SMV configuration, the vessel passes posterior to the vein and is encountered only after the anterior SMV surface has been safely developed. The periportal variant described here represents a qualitatively distinct third configuration: the RCA originates so close to the SMV that no intervening tissue plane exists, and the vessel contacts and visibly indents the anterior SMV wall directly at its origin. This means the vessel is encountered at the very first step of central dissection, before any anatomical landmark has been established, without tactile or visual warning. 

During laparoscopic CME, the standard medial-to-lateral approach develops the plane anterior to the SMV as the primary landmark. Alsabilah et al. (2017) emphasized that inadvertent traction by the first assistant is a well-recognized cause of rupture of the fragile veins in this region, particularly the gastrocolic trunk of Henle [12,14,17,18]. A periportal RCA pressing on the SMV wall would be directly exposed at the earliest stage of this dissection, before any tactile warning of unusual tissue resistance could be appreciated through laparoscopic instruments.

The aberrant MCA and its implications

The concurrent aberrant MCA-arising from the ventral-left SMA surface and immediately bifurcating parallels what Kato et al. (2026) classify as an independent-origin MCA (type present in 19% of their 591-patient CTC-A cohort) [10,19]. Their analysis showed that independent-origin MCA was consistently associated with shorter D3 distances (ICA root to right MCA root: 22.5 vs. 27.5 mm when RCA present, p=0.012; 16.5 vs. 20.5 mm when RCA absent, p<0.001), potentially making central dissection less technically demanding from a length perspective.

The left MCA branch running along the inferior pancreatic border in the present case is anatomically consistent with the accessory MCA (aMCA) extensively described in the literature. In the classification of Kato et al. (2026), this immediate bifurcation into right and left branches qualifies as an independent-origin MCA, which was present in 19% of their cohort and associated with shorter D3 dissection distances [10]. Kato et al. found aMCA in 38.1% of their cohort, with approximately 23% of aMCA origins situated dorsal to the pancreas, requiring posterior pancreatic mobilization for safe CVL. The rare occurrence of drainage of a colic vein into the splenic vein was documented. The left MCA branch vein drained to the IMV in 17.8% and to the splenic vein in 1.2% of cases [10]. The complete absence of a common trunk producing divergent drainage was reported in combination with a periportal RCA in the present case. A pooled prevalence of approximately 25% for aMCA is reported in a meta-analysis by Cheruiyot et al. (2021), with aMCA most commonly arising from the SMA (~88%) and its accompanying vein draining predominantly into the IMV [15,20]. In the present case, however, the left MCA branch vein drained into the splenic vein rather than the IMV, representing an additional departure from expected anatomy.

The right MCA branch ascending toward the hepatic flexure creates a potentially overlapping supply territory with the periportal RCA. When the RCA is absent, the right MCA branch typically compensates as the primary supply to the ascending colon and hepatic flexure [8,21]. Here, both vessels are present and supply overlapping territories, which is surgically relevant: ligation of the periportal RCA alone may not suffice for adequate devascularization of the right colon specimen during CME.

Anomalous middle colic venous drainage

The MCV is almost universally present (97-100% across large series) but shows marked variation in number and drainage destination [6,12,17,22]. Maki et al. (2016) characterized MCV tributaries in 331 patients by 3D CT angiography and found overall drainage to the SMV in 62.5%, GCT in 29.3%, IMV in 4.8%, splenic vein in 2.7%, and jejunal vein in 0.6% [23]. Kato et al. (2026), using higher-resolution CTC-A with branch-level granularity, found that when MCA branches drained separately, the right branch vein favored the GCT (70.4%), while the left branch vein favored the SMV (71.9%) and IMV (17.8%), with splenic vein drainage in 1.2% [10].

The absence of a common MCV trunk in the present case, with the left tributaries draining to the splenic vein and right tributaries draining independently to the SMV, is a bifid pattern without anatomical convergence. Chrysikos et al. (2018) reported a rare case of MCV draining to the splenic vein encountered during right hemicolectomy, emphasizing the need for surgeons to recognize this variant to avoid uncontrolled bleeding [13]. Kato et al. (2026) note that surgeons performing MCA ligation in right hemicolectomy should plan to control either (i) the right MCV branch arising from the SMV if a common trunk exists or (ii) the right-branch vein entering the GCT when branches drain separately [10]. Neither of these standard strategies addresses a pattern where the left MCV tributaries drain to the splenic vein, and no common trunk exists.

Practically, failure to identify the splenic vein tributary preoperatively would leave an uncontrolled venous channel communicating with the splenic venous system. Division of the left transverse mesocolon without ligating this tributary could produce persistent hemorrhage from the splenic vein, a vessel remote from the standard right colonic dissection field and considerably more difficult to control laparoscopically.

Embryological basis

The SMA and its colic branches develop from the vitelline arterial plexus during the fifth to eighth weeks of embryogenesis, with progressive regression of plexiform channels and establishment of the dominant arterial pattern. The SMV forms from the right vitelline vein. The normal spatial separation between the SMA (posterior-left) and SMV (anterior-right) is established during the 270° counterclockwise midgut rotation. Aberrant persistence of ventral plexiform SMA-SMV connections during this process could plausibly give rise to an RCA that retains a ventral origin and crosses directly over the SMV anterior wall [10].

The co-occurrence of anomalous MCA origin, aberrant MCA bifurcation pattern, and bifid MCV drainage without a common trunk in the same specimen suggests a broader disturbance of vascular morphogenesis in the right mesocolon rather than an isolated single-vessel anomaly. In their reproducibility analysis, Kato et al. (2026) observed that MCA type disagreement frequently co-occurred with RCA type shifts and changes in aMCA presence, suggesting that these variables may be jointly determined during embryogenesis rather than being independent variables [10]. The present case is consistent with this hypothesis.

Comparisons with similar case reports

Case reports of unusual RCA origins are infrequent but instructive. Oliveira et al. (2020) described an RCA arising from the right branch of the MCA, found in 36% of Haywood et al.'s (2016) cadaveric series and 2.5% of Haywood et al.'s (2017) systematic review, emphasizing the terminological confusion surrounding vessels not arising directly from the SMA [21,24]. Jongue et al. (2018) described an unconventional RCA origin in a case report highlighting the difficulty of applying standard classifications when the vessel's proximal course is ambiguous [25].

None of these reports involves a ventral SMA origin with periportal SMV compression, nor do any involve the concurrent bifid MCV drainage described here. The Haywood et al. (2016) cadaveric study noted that the RCA originating from the right branch of the MCA was found in 36% of cases, making it a more common variant than direct SMA origin (32%) in that series, and called for improved terminological precision in classifying right colic vessels [24]. Our case adds a qualitatively distinct anatomical configuration that current terminology does not capture.

Surgical implications

The combined vascular anomalies described carry significant surgical implications that deserve careful consideration. Preoperative high-resolution (0.5 mm) triphasic CTC-A, as described by Kato et al. (2026), should be considered mandatory before laparoscopic CME/D3 lymphadenectomy, as its curved planar reformation technique along the SMA centerline can reveal ventral vessel origins and periportal relationships that would remain invisible on standard axial imaging [10]. During surgery, the anterior SMV surface represents the primary danger zone: the standard medial-to-lateral CME approach uses this surface as its primary dissection plane, and a periportal RCA compressing the anterior SMV wall would be encountered immediately and without warning at the earliest stage of central dissection, directly after ligation of the ileocolic pedicle. Regarding arterial control, the overlapping supply territories of the periportal RCA and right MCA branch mean that both vessels must be individually identified and ligated to achieve adequate devascularization of the right colon specimen; intraoperative near-infrared fluorescence may assist in confirming complete devascularization after sequential pedicle ligation. Venous control is equally demanding: the absence of a common MCV trunk and the divergent drainage of left tributaries to the splenic vein and right tributaries directly to the SMV require each channel to be independently identified and controlled, making preoperative venous mapping essential. Most critically, division of the mesocolon without prior identification of the splenic vein tributary risks uncontrolled hemorrhage from a vessel that is neither routinely ligated nor easily accessible through the standard right-sided laparoscopic field, and failure to anticipate this variant may necessitate emergency conversion to open surgery [10].

Limitations

This report is based on a single formalin-fixed cadaveric specimen, which precludes the determination of the true prevalence of this vascular constellation. Formalin fixation may have altered tissue consistency and spatial relationships, limiting precise morphometric measurement. Histological confirmation of vessel identity was not performed, and the donor's unknown clinical history precludes entirely excluding an underlying vascular pathology. As this was a cadaveric study, no radiological correlation or intraoperative validation was possible, and the hemodynamic significance of periportal SMV compression cannot be assessed in a fixed specimen. The surgical implications discussed are therefore extrapolated from anatomical findings and supported by the existing literature rather than confirmed by direct clinical observation.

## Conclusions

We report a previously undescribed combination of right colonic vascular anomalies: (1) a right colic artery arising from the ventral SMA surface with a periportal course producing visible indentation of the anterior SMV wall; (2) an aberrant MCA arising from the ventral-left SMA surface with immediate bifurcation into right and left branches, the latter following a retropancreatic course; and (3) bifid middle colic venous drainage without a common trunk, with left tributaries draining to the splenic vein and right tributaries draining independently to the SMV. This case illustrates that right colonic vascular anomalies may occur in complex, previously undescribed combinations that simultaneously affect the arterial and venous systems. Existing classification systems do not capture the SMA surface of origin as a distinct variable and would not predict the periportal relationship described here. The true prevalence of this vascular constellation remains unknown and warrants large-scale prospective CTC-A studies. The co-occurrence of three concurrent anomalies suggests a possible shared embryological basis requiring further investigation. Existing classification systems should be revised to incorporate the SMA surface of origin as an explicit variable, thereby standardizing reporting in future anatomical and surgical studies.

As minimally invasive right hemicolectomy with CME and D3 lymphadenectomy becomes the standard of care, the SMV dissection corridor demands the same preoperative anatomical precision as hepatopancreatic surgery. Preoperative high-resolution CTC-A should be considered mandatory in centers performing laparoscopic D3 lymphadenectomies. This case also strengthens the argument for a revised, internationally agreed classification of right colonic vascular variants that explicitly records the SMA surface of origin and the periportal relationships of each variant.
